# Optimization of oil yield of *Pelargonium graveolens* L'Hér using Box-Behnken design in relation to its antimicrobial activity and in silico study

**DOI:** 10.1038/s41598-023-47170-0

**Published:** 2023-11-14

**Authors:** Sanagik Sabry Abu El Wafa, Ahmed A. El-Ashmawy, Hanaa A. H. Kassem, Ibrahim H. Eissa, Mohammed Abu-Elghait, Nermin A. Younis, Inas Y. Younis

**Affiliations:** 1 Pharmacognosy Department, Faculty of Pharmacy, Ahram Canadian University, Giza, 12451 Egypt; 2 Medicinal and Pharmaceutical Chemistry Department, Pharmaceutical and Drug Industries Research Institute, National Research Centre, Giza, 12622 Egypt; 3 Pharmacognosy Department, Faculty of Pharmacy, Cairo University, Cairo, 11562 Egypt; 4 Pharmaceutical Medicinal Chemistry and Drug Design Department, Faculty of Pharmacy (Boys), Al-Azhar University, Cairo, 11884 Egypt; 5 Department of Botany and Microbiology, Faculty of Science, Al-Azhar University, Cairo, Egypt

**Keywords:** Microbiology, Plant sciences, Chemistry

## Abstract

*Pelargonium graveolens* L'Hér is an important species of genus *Pelargonium* with an economic value. The unique rose scent of its oil is used in perfume and cosmetic industry. The oil is characterized by the presence of citronellol, geraniol and rose oxide. Fresh aerial parts of *P. graveolens* at GC–MS analysis of four seasons revealed that autumn constituted the highest yield of the oil. For the first time, optimization of the yield of extracted oil of *P. graveolens* was performed employing 3-level Box-Behnken design using 3-factors. The GC–MS analysis of the essential oil was performed for the 17-runs. The optimized extraction of the oil was performed employing numerical optimization and studied for antimicrobial, Minimum Inhibitory Concentration (MIC) and biofilm inhibitory activities. The 3 factors followed rank (plant material amount > water volume > NaCl percent in water), in their magnitude of effect on increasing yield of the oil. Increasing the plant material amount increased the yield of the oil by 6-folds compared to NaCl percent in water. The optimized yield of oil (4 ml) was obtained from extraction criteria (150 g of plant, 750 ml of water and 3.585% (26.85 g) of NaCl). Computational docking was performed to overcome the multi-drug resistant *Gram*-negative bacilli targeting undecaprenyl pyrophosphate synthase (UPPS). The optimized oil exhibited a promising inhibitory activity against Gram-negative bacteria (*K. pneumonia* and *P. aeruginosa*) with significant antibiofilm action (*P* < 0.05). Moreover, it exerted a synergistic effect when combined with various antibiotics (Cefoxitin, Cloxacillin, Oxacillin and Vancomycin) against MRSA clinical strains.

## Introduction

*Pelargonium graveolens* L'Hér (*P. graveolens*), which is known as sweet-scented geranium is a valuable industrial plant that belongs to family Geraniaceae. It’s an aromatic and herbaceous shrub, up to 1 m high. Leaves are carved with small, pink flowers. It’s native to "South Africa and widely cultivated in Russia, Egypt, Morocco, Japan, Algeria, Congo, Central America and Europe^1^". The beautiful pink color flower has unique rose scent with antioxidant, anti-inflammatory, antimicrobial, hypoglycemic activities^2^.

On industrial scale, *pelargonium* oil has gained a special interest mainly in skin-care cosmetic industry and perfumes. Citronellol, geraniol, linalool, citronellol acetate and *p*-menthone were the main volatile ingredients previously identified in Algerian *pelargonium*^3^. Citronellol or dihydro-geraniol, is a natural acyclic monoterpenoid that is widely incorporated in cosmetics and rose-based skin care products especially for its unique floral scent with antimicrobial and anticonvulsant activities^4^.

Unlike the modern method for extraction of essential oils, hydro-distillation is a traditional method of oil extraction where hot water is employed as an ecofriendly media for extraction of volatile components with high yield^5^. Interestingly, the geographical origin, season of collection morphogenetic, and environmental factors highly influence the volatile constituents and must be considered in metabolite profiling of essential oils. Seasonal variation is an important factor that greatly affect oil composition and yield as reported in Greece *pelargonium graveolens*^6^.

Design of experiments (DoE) is the systematic statistical approach employed for conducting experiments with a minimum number of trials or experiments. It involves selecting and controlling various variables that may affect the outcome of an experiment and arranging them into a series of tests or trials. The goal of DoE is to identify the important variables and their interactions, and optimize the experimental conditions to achieve the desired outcome. DoE is widely used in scientific research, product development, process improvement and quality control. It provides a good manipulation of various input factors to get the desired output or to improve the result. Interestingly, DoE allows an excellent control of multiple input parameters, or "factors", such as temperature and concentration with the assessment to explain the conditions at which the product attributed. Importantly, with fewer experimental repetitions DoE can reduce time consumption and decrease the development cycles in manufacturing^7^.

Using DoE, Response surface methodology (RSM) can efficiently analyze the effects of various factors and find the optimal conditions for the desired outcome. Box–Behnken design (BBD) is a type of RSM design that can effectively determine how the responses depend on the factors under study^8^. Moreover, with a minimum number of runs, BBD can provide detailed information on how the experimental variables influence the responses and the overall experimental error.

Methicillin-resistant *Staphylococcus aureus* (MRSA) is Gram-positive bacterium with a global prevalence. Recently, it had emerged and spread worldwide with arsenal of virulence factors and was responsible high rate of mortality in elderly care centers according to United State cohort study^9–12^. Vancomycin is the conventional treatment of MRSA infection. However , the misuse of antibiotics leads to outbreak of resistance bacteria and serious side effects such as nephrotoxicity, untreatable Gonorrhea, fungal infections, and soft tissue infection^13–15^.

Peptidoglycan in bacterial cell wall is a promising target for discovery of a lead broad spectrum antimicrobial drug. Undecaprenyl pyrophosphate synthase (UPPS) is an important enzyme involved in the peptidoglycan cell wall synthesis and catalyzes the transformation of farnesyl pyrophosphate **I** (FPP) to undecaprenyl pyrophosphate (UPP)^16,17^. Most Undecaprenyl Pyrophosphate Synthase Inhibitors (UPPSIs) are bisphosphonate derivatives. This class of derivatives include BPH-629 (**II**)^18^, Farnesyl thiopyro-phosphate (III)^19^**, IV**^20^, BPH-1290 (**V**)^21^, and **VI**^22^.

Extensive literatures discussed the composition of *pelargonium* oil with respect to different geographic origin^23^. However, to the best of our knowledge, no comprehensive investigation regarding seasonal variation and no data is available concerning the optimization of the yield of the extracted oil during four seasons using Box-Behnken design and its correlation to its antimicrobial potential. Docking study using undecaprenyl pyrophosphate synthase was selected as an active site for citronellol.

Therefore, the aim of the present study was to optimize the yield of the essential oil of Egyptian *P. graveolens* by using RSM, as well as, to investigate the anti-microbial activity of the optimized extract of *P. graveolens* essential oil against MRSA. Employing BBD, the effects of plant material amount, water volume, NaCl percent in water and their effect on quality of the oil were studied. Moreover, the floral scent compositions were evaluated during the seasonal climatic changes by gas chromatography–mass spectrometry analysis (GC–MS).

## Material and methods

### Plant material

Fresh *P. graveolens* aerial parts were hand-picked in the early morning during spring(in March), summer(in July), autumn (in October) and winter (in January) from "the Experimental Station of Faculty of Pharmacy, Cairo University" , after permission from "Agricultural Research Center, Giza, Egypt" allocated in "9, Cairo University Road, Oula, Giza District, Giza Governorate". It was picked in compliance with the national guidelines to preserve the volatile scent of *pelargonium*. It was gratefully identified by Mrs. Therese Labib, consultant at "Ministry of Agriculture and the former director of El-Orman Botanic Garden". A voucher specimen was deposited in the herbarium of "Pharmacognosy department, Faculty of Pharmacy, Cairo University, Cairo, Egypt" with code number 2–05–2020.

### Chemicals and materials

All chemicals were purchased from Sigma Aldrich, USA while, antibiotic standards including; cefoxitin, oxacillin, cloxacillin and vancomycin were from Thermo Scientific™ Oxoid™, USA. All procedures were approved according to National guidelines of Faculty of Pharmacy, Cairo University and institutional biosafety committee No: MP (2693).

### Extraction of essential oil

Hydro-distillation of fresh aerial parts of P. graveolens using a well-established apparatus (Clevenger-type ) at 100 °C. The essential oil was light yellow and had a characteristic rose-like odor. The golden yellow oil was dehydrated over anhydrous Na2SO4 and stored in brown bottle at 4 °C for further analysis. The same procedure was applied during summer, winter, spring, autumn and the oil obtained from the four seasons was analyzed by GC–MS. The comparison was established between oil compositions of 4 seasons. The season with the highest percentage of citronellol and the yield of oil was selected for optimization.

### GC–MS analysis

#### GC–MS analysis of essential oil

GC/MS analysis was established using Shimadzu GCMS-QP2020 (Tokyo, Japan) equipped Rxi-SVOC ms GC Capillary Column (30 m × 0.25 mm i.d. × 0.25 µm film thickness) according to the method previously described in Mohsen et al., 2020. The split–splitless injector was used with an initial isothermal column temperature at 45 °C for 2 min then the temperature raised gradually to 300 °C at a rate of 5 °C/min^24^.

#### Peak identification

The comparison of the obtained mass-spectra and retention indices with the data available in the NIST, and WILEY library database were performed. Kovats retention index as an efficient mathematical tool (KI) was also used relative to n-alkanes (C6-C20).

### Optimization of the method of extraction of the essential oil

#### Design of experiment (DoE)

The optimization design was carried out using BBD “3-factor, 3-level based on 3^3^-factorial designs" employing “Design Expert version v.8 software”. The design was based on analysis of 17-runs involving 5-center points. The studied 3-independent variables (independent factors), 8-dependent variables (dependent responses) and their actual and coded levels are illustrated in Table 1, while the BBD and the measured responses values are explained in Table 2. In the study, 3 factors that can affect the quantity of yield and the main active constituents were chosen. The 3 factors, each of them was studied at 3-levels, were: i) The plant material amount (50 g,100 g and 150 g), ii) The water volume (250 ml, 5ooml and 750 ml) and iii) The NaCl percent in water (1.5%, 3% and 4.5%). The oil was extracted in this study by hydro-distillation method, after that GC–MS analysis was performed for each run.

**Table 1:** Box-Behnken Design (BBD) variables were evaluated at both their actual and coded levels.

	Levels used		
	Low (− 1)	Middle (0)	High (1)
*Independent variables*			
X1: plant material amount (gm)	50 g	100 g	150 g
X2: water volume (ml)	250 ml	500 ml	750 ml
X3: NaCl percent in water (%)	1.5%	3%	4.5%
*Dependent variables*			
Y1: yield of oil (ml)			
Y2: citronellol (Area %)			
Y3: geraniol (Area %)			
Y4: Gamma-Eudesmol (Area %)			
Y5: citronellol acetate (Area %)			
Y6: I-Menthone (area %)			
Y7: linalyl acetate (area %)			
Y8: rose oxide (area %)

**Table 2:** Response surface design and corresponding response values of the Box-Behnken.

Std. order	Run	X1 Plant material amount (gm)	X2 Water volume (ml)	X3 NaCl percent in water (%)	Y1 Yield of oil (ml)	Y2 Citronellol (Area%)	Y3 Geraniol (Area%)	Y4 Gamma-Eudesmol (Area%)	Y5 Citronellol acetate (Area%)	Y6 I-Menthone (Area%)	Y7 Linalyl acetate (Area%)	Y8 Rose oxide (Area%)
11	1	100 g	250 ml	4.5% (11.25 g)	0.2	36.09	9.29	9.64	8.11	6.28	3.14	1.54
14	2	100 g	500 ml	3% (15 g)	0.25	37.1	9.77	10.29	7.73	5.72	3.02	1.39
2	3	150 g	250 ml	3% (7.5 g)	0.3	38.04	13.83	8.55	8.56	6.32	2.9	1.65
12	4	100 g	750 ml	4.5% (33.75 g)	0.3	42.08	12.87	13.13	9.65	5.47	2.97	1.27
4	5	150 g	750 ml	3% (22.5 g)	0.4	48.76	11.46	9.78	10.19	6.12	2.43	2.18
10	6	100 g	750 ml	1.5% (11.25 g)	0.25	40.14	16.28	10.45	11	6.6	2.76	1.46
17	7	100 g	500 ml	3% (15 g)	0.25	38.23	10.12	9.15	7.81	6.22	3.13	1.55
8	8	150 g	500 ml	4.5% (22.5 g)	0.35	39.57	8.63	9.67	8.75	6.56	2.57	1.87
13	9	100 g	500 ml	3% (15 g)	0.25	38.31	12.04	10.36	8.29	6.07	2.87	1.64
15	10	100 g	500 ml	3% (15 g)	0.25	38.21	8.79	9.84	8.88	6.05	2.99	1.6
6	11	150 g	500 ml	1.5% (7.5 g)	0.3	38.64	17.96	11.03	8.53	6.86	4.36	1.03
5	12	50 g	500 ml	1.5% (7.5 g)	0.2	35.79	14.2	10.23	8.41	5.67	2.94	1.5
16	13	100 g	500 ml	3% (15 g)	0.25	38.46	17.81	10.76	9.74	6.98	3.54	1.04
7	14	50 g	500 ml	4.5% (22.5 g)	0.2	36.8	18.32	12.88	8.4	6.65	3.55	0.94
1	15	50 g	250 ml	3% (7.5 g)	0.1	34.4	11.71	9.58	7.83	6.44	3.64	1.38
9	16	100 g	250 ml	1.5% (3.75 g)	0.2	35.8	9.98	9.72	8.21	6.56	2.68	1.37
3	17	50 g	750 ml	3% (22.5 g)	0.25	35.32	21.1	11.4	9.71	6.18	3.41	0.98

### In silico docking studies

#### Protein preparation

The crystal structures of the selected protein i.e., undecaprenyl pyrophosphate synthase “PDB ID: 1V7U, resolution: 2.35 Å", was obtained from the global source of biological macromolecules, Protein Data Bank (https://www.rcsb.org). The undecaprenyl pyrophosphate synthase protein was prepared after removal of any water molecules. The resulting chain was retained along with the co-crystallized ligand (Farnesyl diphosphate). The initial protonation of the selected protein chain was established using the following setting: “The electrostatic functional form was GB/VI with a distance cut-off of 15 Å". To improve the docking performance the dielectric constant was set at 2 with an 80 dielectric constant of the selected solvent. Van der Waals functional form was set 800R3 with a distance cut-off of 10 Å. The structural optimization was maintained by the energy minimization of the selected protein using “Hamiltonian AM1 implanted in Molecular Operating Environment" (MOE 2019 and MMFF94x) “Merck molecular force field". The validation of docking protocol was established by redocking, and calculation of root mean square deviation (RMSD). The accurate identification of the binding site was performed as the residues positioned within the 5 Å distance from the perimeter of ligand^25^.

#### Ligand preparation

2D structures of citronellol and its ligand (Farnesyl diphosphate) were carefully drawn using “Chem Bio-Draw Ultra 14.0 and saved in MDL-SD file format". The protonation of the 3D structures of citronellol were established followed by energy minimization using MM2 force-field and 10,000 iteration steps of 2 fs. Finally, the establishment of the docking studies was performed using the conformationally optimized ligands^26^.

#### Docking setup and validation of docking protocol

Molecular Operating Environment (MOE, version 2019) was established to perform the protein–ligand docking study. The docking protocol was validated by redocking of the co-crystallized reference ligand against the isolated protein pocket. Followed by comparison of Root mean square deviation (RMSD) of the re-docked ligand poses with its corresponding reference ligand structure^27^. Importantly, the docking setup of citronellol was developed according to the previously described protocol followed the validation step. Structurally, 30-docked solutions were generated for each docking run, using ASE for scoring function and rigid receptor for refinement. “Discovery Studio (DS) 4.0." as a comprehensive software suite tool used to visualize the docking results. While, analysis of results was based on the comparison of the docking score of citronellol with the re-docked reference molecule^28,29^.

### Biological studies

#### Strains and culture conditions

*Pseudomonas aeruginosa, Klebsiella pneumonia, Streptococcus mutans, Candida albicans,* and *Staphylococcus aureus* (DNF1 and DNF2) clinical isolates were used for antimicrobial, antibiofilm, and drug combination assays. The Vitek2 system recognized all of these isolates. Susceptibility profiles of these strains were evaluated manually and by using the VITEK®2 automated system (BioMerieux) and selected as multidrug-resistant strains. MSSA ATCC 25,923 strain was also utilized.

#### Anti-microbial activity assay

The antibacterial activity of the optimized oil was performed using Agar well diffusion assay. Muller Hinton agar (MHA) (Oxoid, USA) and Tryptic soy agar (TSA) (Oxoid, USA) media were utilized to assess the growth of bacterial and yeast strains. After 24-h, bacterial suspension was inoculated in autoclaved media with a turbidity of 0.5 McFarland standard. Then evenly, they were dispensed into Petri dishes. By using a sterile cork-borer, a well with a diameter of 8 mm was punched, and 100 μl of the optimized oil was inoculated into the well and then incubated at 37 °C for 24 h. Using a digital caliper after incubation, the growth inhibition zone was measured. The zones' diameters were measured in millimeters. The result was documented in accordance with the CLSI guidelines^30^.

#### Minimum inhibitory concentration (MIC) detection

Assessment of the MIC of the oil against test organisms utilizing a microdilution assay using microtiter plates (MTP) was conducted according to the previous approach^31^, with minor modifications. In brief, a twofold serial dilution of the extract from 200 to 0.78 µl/ml w/v was distributed in a 96-well plate (SPL, Korea) and added to the specific microbial cultures inoculated with overnight cultures of the test organisms then the plates were incubated at 37 °C with shaking at 120- rpm for 24- hours. According to the Clinical and Laboratory Standards Institute (CLSI) guidelines^30^, the MIC was determined as the lowest dose of the oil extract that inhibited the test organisms comparable to positive and negative controls using a microplate reader (STATFAX, USA) with a O.D of 620 nm.

#### Drug combination assay

Many bacterial infections are treated with drug combinations. According to the docking results of this study, we tested a combination of the tested oil and the antibiotics viz., Oxacillin, Cefoxitin, Cloxacillin and Vancomycin against MRSA strains. First, the activity was determined using a disc diffusion assay on MHA media (Oxoid, USA) with antibiotic discs Cefoxitin, Oxacillin, Cloxacillin and Vancomycin (Oxoid, USA) alone and in combination with the tested oil. The fractional inhibitory concentration index (FIC) was calculated using a microdilution assay with various concentrations of the tested oil in conjunction with the antibiotics against MRSA^32^. FIC value for the tested oil was calculated using the following formula:

$$ {\text{FICI }} = \, \Sigma {\text{FIC}} = {\text{FIC }}\left( {{\text{antibiotic}}} \right) + {\text{FIC }}\left( {\text{Pelargonium oil}} \right). $$

where FIC (antibiotic) = MIC of antibiotic in combination/MIC of antibiotic alone.

FIC (Pelargonium oil) = MIC of Pelargonium oil in combination/ MIC of Pelargonium oil alone.

When FICI ≤ 0.5, the combination is said to be synergistic, when FICI ≥ 0.5–1, the combination is additive, when FICI ≥ 1.0 ≤ 4.0, the combination was indifferent and when value of FICI exceeds 4.0, the combination was said to be antagonistic^33^.

#### Biofilm inhibition assay

MTP technique was performed against *S. aureus* MRSA identified as a potent biofilm forming clinical isolate, *P. aeruginosa*, and *C. albicans* to test the capability of the essential oil to inhibit or diminish the biofilm development of bacteria and yeast. The biofilm experiment from the previous study^34^ was modified somewhat. Briefly, gradient concentrations of the oil were added to MTP containing TSB Medias supplemented with 1% glucose. The test organisms were diluted 1:100 in TSB and put onto MTP for 48 h at 37 °C. Prior to moving planktonic cells from the plates, the growth density (OD620nm) was determined after the incubation period. The contents of the wells were then removed without disturbing the established biofilms and then washed with phosphate buffered saline (PBS) pH 7.4 3-times, and then the biofilm fixed with 200μL of 95% methanol for 10 min. Each well filled (200μL) with crystal violet (0.3% w/v) and incubated for 15-min at room temperature. Finally, the plates washed with distilled water and acetic acid 30% was added to the wells for quantitative detection of biofilm formation. The absorbance measured at O.D_540nm_ by microplate reader (STATFAX-USA). The results documented by comparing the treated wells with untreated controls^35^.

#### Statistical analyses

3-realistic duplicates were obtained for each assay, and all results are the averages of 3-independent experiments. Student's t-tests were used to examine differences between a sample and its corresponding control. If the *P-* value was less than 0.05, the difference was significant.

## Results and discussion

### GC–MS profiling of the volatile scent of *Pelargonium* oil

**Figure 1:**
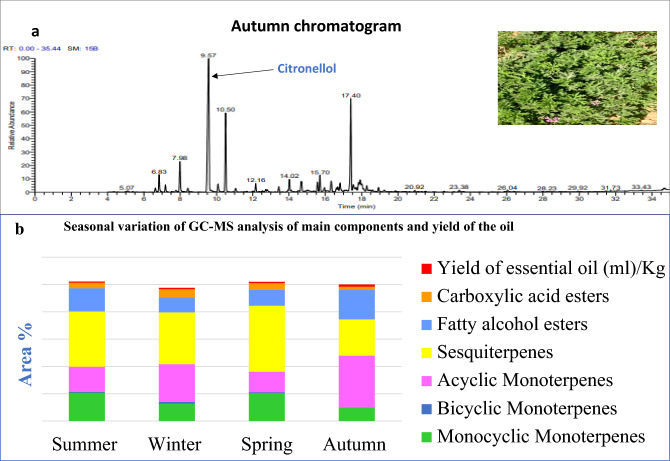
(**a**) GC–Ms of *pelargonium graveolens* oil chromatogram in autumn. (**b**) Seasonal variation of the main volatile components of *pelargonium graveolens* oil.

GC–MS analysis was carried out on the essential oil obtained from the four seasons. Seasonal variation was studied to compare between the different seasons in the quantity of the yield of the oil and active constituents and to determine which season contains the highest quantity. Figure 1 illustrates that the highest quantity of yield and active constituents of the oil was obtained in autumn. Box Behnken design was performed via 17- runs of GC in autumn for optimization of the yield. BBD is established at three levels “− 1, 0, 1” and performed for 8-variables^36^. BBD has the power to build sequential designs without model fit detection, and the prediction of quadratic model parameters with the lowest or highest levels of all factors^8^.

### Experimental design

The independent variables were selected based on previous reported outcomes^37,38^.The 3-independent factors used in this design were (plant material amount, water volume, NaCl percent in water). It was reported that, NaCl didn’t influence the quality of the oil but, it significantly improves the oil yield^39^ and NaCl percent used in many researches was ranging from 0.5% to 7%. So, the effective amount of sodium chloride in extraction of oil is unclear^37–40^. In addition, the selection of the specified amount of plant material and water volume was highly variable in literature^41,42^.

#### Model validation

As illustrated in Table 2, each row represented a single run, with its responses (Yield (Y_1_), Citronellol (Y_2_), Geraniol (Y_3_), gamma-Eudesmol (Y_4_), Citronellol acetate (Y_5_), I-Menthone (Y_6_), Linalyl acetate (Y_7_) and Rose oxide (Y_8_)) and the whole table is defined as the BBD matrix. Meanwhile, Table 3 summarizes the sequential model sum of squares, lack of fit tests, and model summary for each response that involved in 4-models (Linear, 2-factor interaction (2FI), Quadratic and Cubic).

**Table 3:** Sequential model fitting for: Yield(Y_1_), Citronellol(Y_2_), Geraniol(Y_3_), Gamma-Eudesmol(Y_4_), Citronellol acetate(Y_5_), I-Menthone(Y_6_), Linalyl acetate(Y_7_) and Rose oxide (Y_8_). * Statistically significant: *p* ˂0.05.

	Yield (Y_1_)		Citronellol (Y_2_)		Geraniol (Y_3_)		Gamma-Eudesmol(Y_4_)		Citronellol acetate(Y_5_)		I-Menthone (Y_6_)		Linalyl acetate(Y_7_)		Rose oxide(Y_8_)									
	P-value	Remarks	P-value	Remarks	P-value	Remarks	P-value	Remarks	P-value	Remarks	P-value	Remarks	P-value	Remarks	P-value	Remarks								
Sequential sum of squares																								
Mean												S				S								
Linear	˂0.0001*	S	0.0005		0.2003		0.0233*		0.0068*	S	0.5899		0.7398		0.2200									
2Fi	0.4347		0.0517*	S	0.0737	S	0.0496*		0.8449		0.3902		0.0961	S	0.0216*	S								
Quadratic	0.4764		0.6297		0.3121		0.0517*	S	0.3410		0.8996		0.5443		0.6592									
Cubic	0.3099	A	0.0082	A	0.9886	A	0.7942	A	0.8666	A	0.4287	A	0.0702	A	0.5397	A								
Lack of fit tests																								
Linear	0.4065	S	0.0075*		0.5490		0.1575		0.8575	S	0.6689		0.0687		0.2956									
2FI	0.3804		0.0158*	S	0.8235	S	0.3130		0.7474		0.6796		0.1103	S	0.6810	S								
Quadratic	0.3099		0.0082*		0.9886		0.7942	S	0.8666		0.4287		0.0702		0.5397									
Cubic		A		A		A		A		A		A		A		A								

	Adj R^2^	Pred R^2^	Remark	Adj R^2^	Pred R^2^	Remark	Adj R^2^	Pred R^2^	Remark	Adj R^2^	Pred R^2^	Remark	Adj R^2^	Pred R^2^	Remark	Adj R^2^	Pred R^2^	Remark	Adj R^2^	Pred R^2^	Remark	Adj R^2^	Pre R^2^	Remark
Model summary statistics source																								
Linear	0.865	0.797	S	0.666	0.441		0.127	− 0.22		0.392	0.048		0.502	0.385	S	− 0.06	− 0.49		− 0.12	− 0.83		0.113	− 0.3	
2FI	0.864	0.678		0.793	0.372	S	0.416	0.191	S	0.626	0.141		0.402	0.042		− 0.04	− 1.01		0.20	− 1.25	S	0.543	0.1	S
Quadratic	0.861	0.420		0.765	− 0.54		0.483	0.556		0.811	0.625	S	0.455	0.107		− 0.37	− 3.97		0.14	− 3.88		0.473	− 0.6	
Cubic	0.892		A	0.972		A	0.121		A	0.738		A	0.190		A	− 0.28		A	0.70		A	0.434		A

Complete sequential model fitting for all responses was summarized in supplementary tables (Table supp.2 (a, b, c, d, e, f, g & h)).

*Statistically significant: p ˂0.05.

S. Suggested.

A. Aliased.

These tests were conducted to determine the model's sufficiency to provide the highest yield. Coefficient of determination (R^2^), adjusted coefficient of determination (R^2 a^) and coefficient of variance (CV%) were determined for the evaluation of the reliability of the model fit. Analysis of variance (ANOVA) was used to statistically examine each factor's effect on individual responses. The lack of fit and sequential model sum of squares tests were applied for each response for linear, 2FI, quadratic, and cubic models.

Table 3 illustrates that the linear model was chosen for Y_1_ and Y_5_ responses according to: (i) The sum of squares analysis showed significant *p*-values (*p* = ˂0.0001 for Y_1_ and 0.0068 for Y_5_), (ii) The lack of fit test showed non-significant p values (*p* = 0.4065, 0.8575 for Y_1_ and Y_5_, respectively), (iii) Maximum adjusted- R^2^ (0.8650, 0.5028 for Y_1_ and Y_5_, respectively) and (iv) Maximum predicted- R^2^ (0.7974, 0.3857 for Y_1_ and Y_5_, respectively).

While, 2FI was the chosen model for Y_2_, Y_3_, Y_7_ and Y_8_ responses according to: (i) The sum of squares analysis showed significant *p*-values (*p* = 0.0517 for Y_2_, 0,0737 for Y_3_, 0.0961 for Y_7_ and 0.0216 for Y_8_), ii) The lack of fit test showed non-significant *p* values (*p* = 0.0158 for Y_2_, 0.8235 for Y_3_, 0.1103 for Y_7_ and 0.6810 for Y_8_), iii) Maximum adjusted-R^2^ ( 0.7932, 0.4165, 0.2053, 0.5434 for Y_2_, Y_3_, Y_7_ and Y_8_ respectively) and iv) Maximim predicted-R^2^ ( 0.3724, 0.1919, − 1.2519, 0.1019 for Y2, Y_3_, Y_7_ and Y_8_) (Table 3).

Quadratic model was chosen for Y_4_ response due to significant *p* value for sum of squares analysis = 0.0517, non- significant lack of fit test = 0.7942, adjusted R^2^ = 0.8119 and predicted R^2^ = 0.6259. On the other hand, all the four models gave non-significant p-value for Y_6_ response (Table 3)^43^.

#### Verification of model adequacy

**Figure 2:**
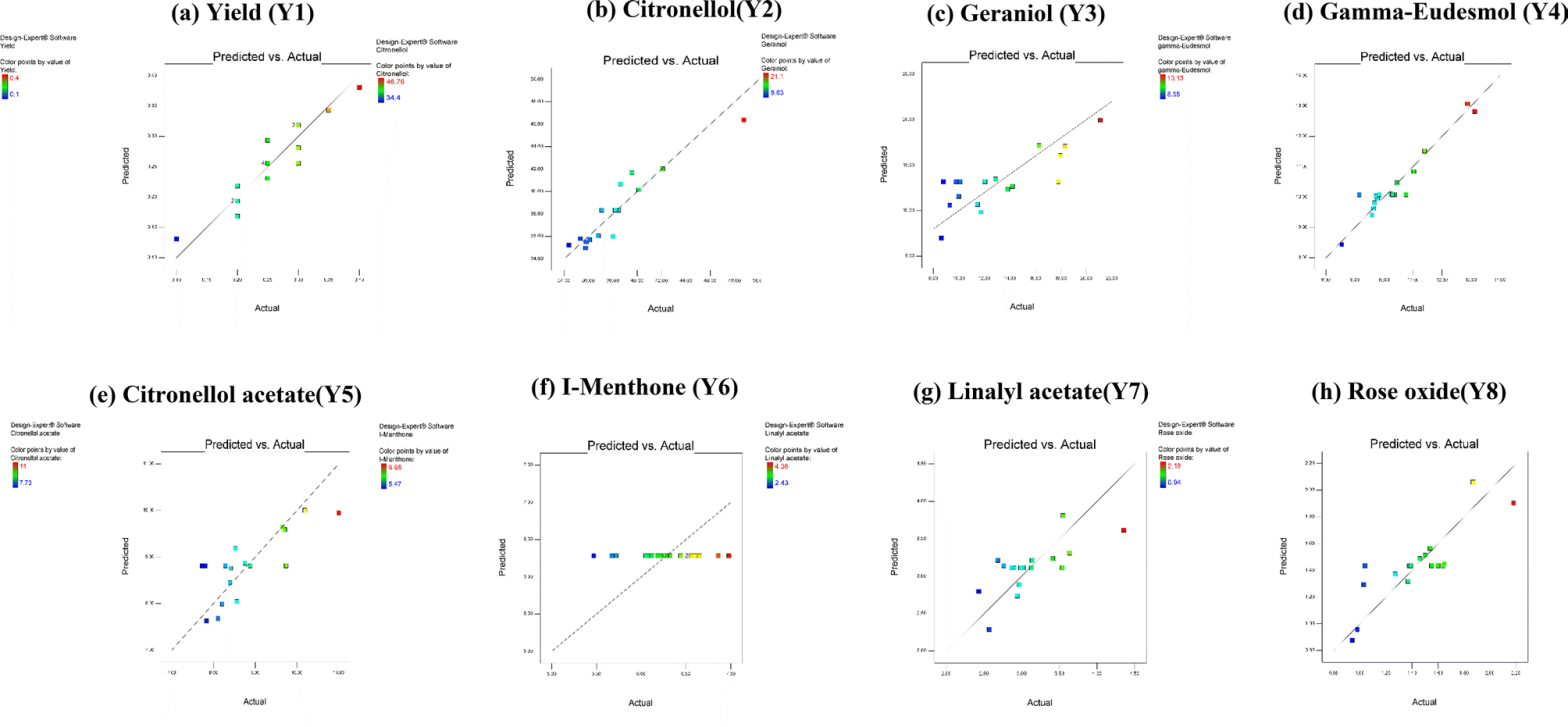
Diagnostic plots for BBD model adequacy. (**a**) Yield, (**b**) Citronellol, (**c**) Geraniol, (**d**) Gamma-Eudesmol, (**e**) Citronellol acetate, (**f**) I-Menthone, (**g**) Linalyl acetate and (**h**) Rose oxide.

Model adequacy is illustrated in Fig. 2, as the predicted values of Y1 to Y8 for each run were calculated and compared to the experimental values. All the points on the scatterplots were very close to the line, which indicated that the experimental results occupied a close arrangement to the predicted values. Therefore, the model showed goodness-of-fit measures and so it will be highly capable to predict the independent-dependent variables’ relationship, except in Fig. 2f the scatterplots points were extremely far from the line and so there was no selected model for I-Menthone(Y_6_) as all the four models are non-significant to it.

#### The yield (Y_1_)

Typically, the coefficient estimates (CE) and *p*-value for each model were indicated in Table 4. The linear coefficients of the effects (Plant material amount (A), Water volume (B) and NaCl percent in water (C)) were highly significant on the yield (*p*˂0.0001). In other words, increasing any of these three factors will increase the yield by the following rank: plant material amount(A) ˃ water volume (B) ˃ NaCl percent in water (C) (CE = 0.075, 0.050 and 0.013, respectively).

**Table 4:** *P*-values and coefficient estimates of the 8-studied responses. * Statistically significant: *p* ˂0.05.

	Y1 yield		Y2 citronellol		Y3 geraniol		Y4 Gamma-Eudesmol		Y5 Citronellol acetate		Y6 I-menthone		Y7 linalyl acetate		Y8 rose oxide	
	*p*-value	Coefficient estimate (CE)	*p*-value	CE	*p*-value	CE	*p*-value	CE	*p*-value	CE	*p*–v	CE	*p*-value	CE	*p*-value	CE
Intercept		0.26		38.34		13.19		10.08		8.81		6.28		3.11		1.43
A-Plant material amount	˂0.0001*	0.075	0.0003*	2.84	0.1373	− 1.68	0.0109*	− 0.63	0.3810	0.21	–	–	0.3063	− 0.16	0.0112*	0.24
B-Water volume	˂0.0001*	0.050	0.0004*	2.75	0.0697	2.11	0.0017*	0.91	0.0010*	0.98	–	–	0.5208	− 0.099	0.9376	− 6.250
C-NaCl percent in water	˂0.0001*	0.013	0.3488	0.52	0.2894	− 1.16	0.0334*	0.49	0.5150	− 0.16	–	–	0.6766	− 0.064	0.6850	0.033
AB			0.0085*	2.45	0.0736	− 2.94	0.5889	− 0.15			–	–	0.7808	− 0.060	0.0608	0.23
AC			0.9792	− 0.020	0.0453*	− 3.36	0.0063*	− 1.00			–	–	0.0170*	− 0.60	0.0098*	0.35
BC			0.5944	0.41	0.6538	− 0.68	0.0330*	0.69			–	–	0.7720	− 0.062	0.4325	− 0.090
A2							0.9470	− 0.018								
B2							0.3855	− 0.24								
C2							0.0099*	0.89								

So, the regression equation will be:

$$ {\text{Yield}} = + 0.{26 } + \, 0.0{\text{75A }} + \, 0.0{5}0{\text{B }} + \, 0.0{\text{13C}} $$

**Figure 3:**
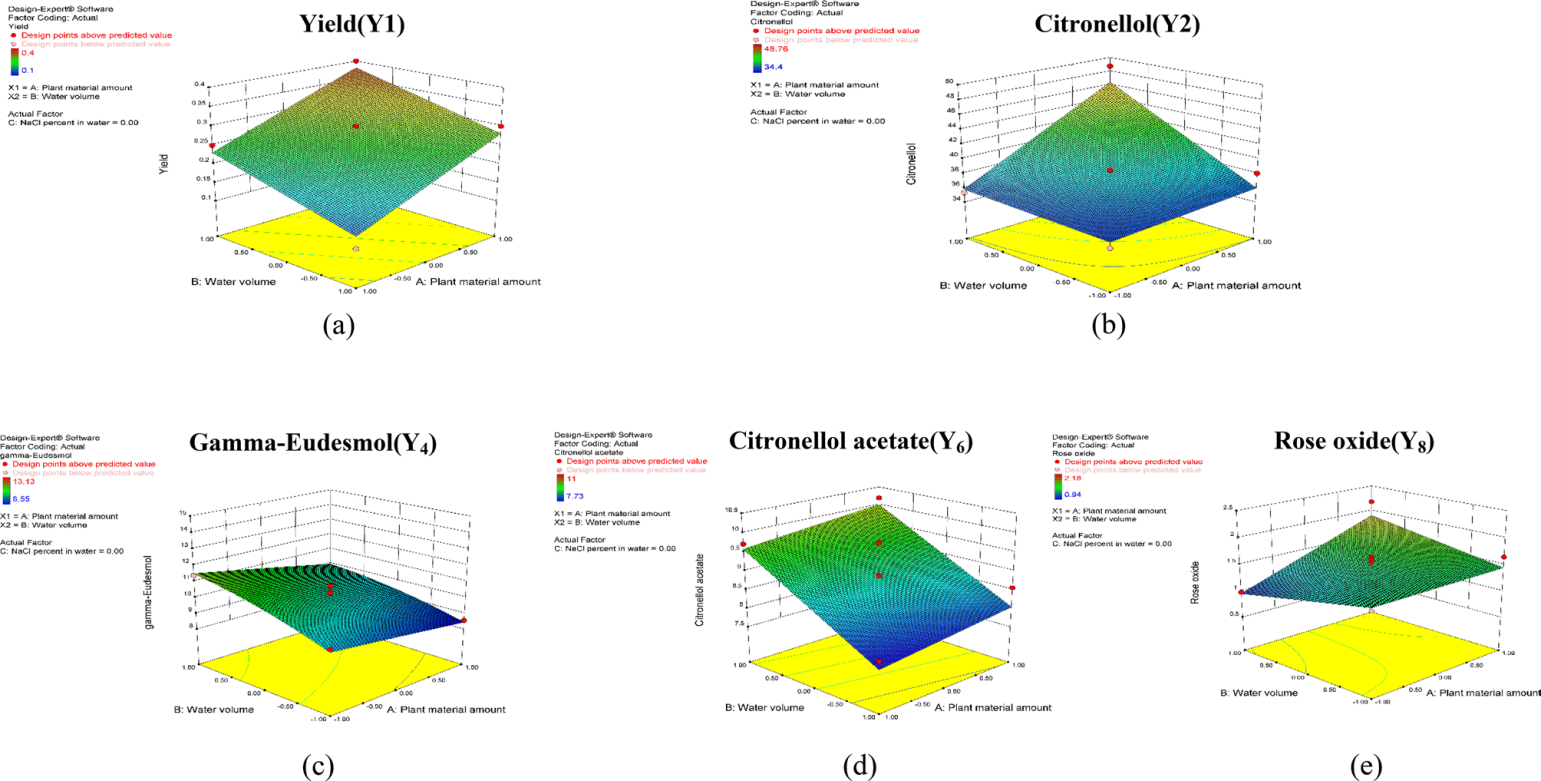
3D response surface plots for (**a**) Yield (Y_1_), (**b**) Citronellol (Y2), (**c**) Gamma-Eudesmol (Y4), (**d**) Citronellol acetate (Y5) and (**e**) Rose oxide (Y8). The Effects of A (Plant material amount), B (Water volume) and their interaction.

The 3D response surface plots illustrated in Fig. 3a show the effects of A, B, C and their interactions on the yield. The significance of the regression equations can be seen from the maxima and minima of the plots^36^. As shown in the Figure, the yield amount increased at the high levels of plant material amount and water volume till a certain limit and then decreased respectively.

#### Citronellol (Y_2_)

It was found that the effects of two linear coefficients (A and B) and one interactive coefficient (AB) were significant on the citronellol. The plant material amount and the water volume were significant (*p*- value = 0.0003 and 0.0004) and were directly proportional to citronellol (CE = 2.84 and 2.75). Only the interactive coefficient (AB) was significant (*p* = 0.0085) and had an increasing effect (directly proportional) to citronellol. Two interactive coefficients (AC and BC) had non-significant effects and their *p*- values = (0.9792 and 0.5944), respectively. While BC had a directly proportional effect (CE = 0.41), AC had an inversely proportional effect (CE = − 0.020). The regression equation will be:

$$ {\text{Citronellol}} = + {38}.{34 } + {2}.{\text{84A }} + { 2}.{\text{75B }} + { 2}.{\text{45AB}} $$

Figure 3b As shown in the Figure, the increase in the plant material and water volume led to an increase in citronellol amount until a certain limit and then any increase in the plant material and water volume will lead to a decrease in citronellol, while NaCl percent had a non-significant effect on the amount of citronellol.

#### Geraniol (Y_3_)

Only the interactive coefficient (AC) was significant on the Geraniol (*p*-value = 0.0453) but it had a decreasing effect (inversely proportional) to the geraniol. The Linear coefficients (A, B and C) and the two interactive coefficients (AB and BC) were non- significant to the geraniol (*p*-value˃ 0.05). While the water volume (B) was the only one that had an increasing effect (directly proportional) on the amount of geraniol.

So, the regression equation will be:

$$ {\text{Geraniol}} = + {13}.{19 }{-}{ 3}.{\text{36AC}} $$

#### Gamma-Eudesmol (Y_4_)

The effect of the linear coefficients (A, B and C), two interactive coefficients (AC and BC) and one quadratic coefficient (C2) were significant to the gamma- Eudesmol (*p*-value ˂ 0.05). Only one interactive coefficient (AB) and two quadratic coefficients (A^2^ and B^2^) are non-significant to the gamma- Eudesmol (*p*-value = 0.5889 for AB, 0.9470 for A^2^ and 0.3855 for B^2^) and also, they had a decreasing effect (inversely proportional) on the amount of gamma-Eudesmol. The equation will be:

$$ {\text{Gamma}} - {\text{Eudesmol}} = + {1}0.0{8 }{-} \, 0.{\text{63A }} + \, 0.{\text{91B }} + \, 0.{\text{49C }} - { 1}.00{\text{AC }} + \, 0.{\text{69BC }} + \, 0.{\text{89 C}}^{{2}} $$

Figure 3c As shown in the figure, the gamma-Eudesmol amount increased at the low level of plant material till certain limit and then decreased respectively. While, the gamma-Eudesmol amount increased at the high level of water volume.

#### Citronellol acetate (Y_5_)

Only the Linear coefficient (water volume (B)) was found to be significant (*p* = 0.0010) and it was directly proportional to the amount of citronellol acetate. While linear coefficient (NaCl percent in water (C)) was non-significant (*p* = 0.5150) and it was inversely proportional to the citronellol acetate.

So, the regression equation will be:

$$ {\text{Citronellol}}\;{\text{acetate}} = + {8}.{81 } + \, 0.{\text{98B}} $$

Figure 3d as shown in the figure, citronellol acetate increased at the high level of water volume, while citronellol acetate increased until certain limit and then decreased at the high level of plant material.

#### Linalyl acetate (Y7)

Only the interactive coefficient (AC) was significant (*p* = 0.0170) and it had a decreasing effect (inversely proportional) on the amount of linalyl acetate (CE = − 0.60). The linear coefficients (A, B and C) and the two interactive coefficients (AB and BC) were non-significant (*p* ˃ 0.05) and they also; they have a decreasing effect (inversely proportional) to the linalyl acetate. So, the regression equation will be:

$$ {\text{Linalyl acetate}} = \, + {3}.{11 }{-} \, 0.{6}0{\text{AC}} $$

#### Rose oxide (Y8)

The linear coefficient (Plant material amount (A)) and the interactive coefficient (AC) were significant to the rose oxide (p-value = 0.0112 for A and 0.0098 for AC) and they had an increasing effect (directly proportional) on the amount of rose oxide. Simplification of the regression equation was presented in the following equation:

$$ {\text{Rose}}\;{\text{oxide}} = + {1}.{43 } + \, 0.{\text{24A }} + \, 0.{\text{35AC}} $$

Figure 3e as shown in the figure rose oxide amount increased at the high level of the plant material and at the low level of water volume, while it decreased at the low level of plant material and the high level of water volume. NaCl percent was non-significant and had no effect on the amount of rose oxide.

**Figure 4:**
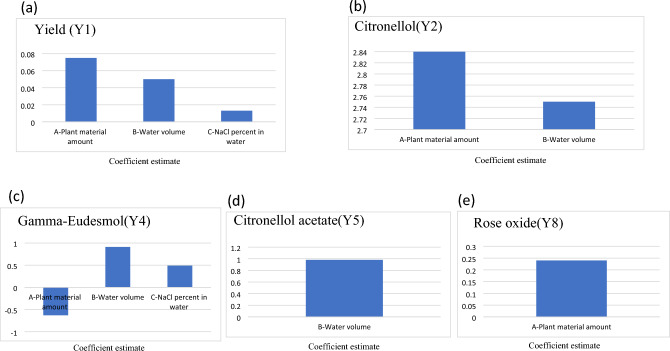
Coefficient estimates of the studied factors on significant effects for: (**a**) Yield, (**b**) Citronellol, (**c**) Gamma-Eudemol, (**d**) Citronellol acetate (e)Rose oxide.

Figure 4 visualizes the coefficient estimates (CEs) for the factors affecting significantly the studied responses. Figure 4a demonstrates that the plant material amount had the highest increasing effect on the amount of the yield compared to water volume and NaCl percent in water. (Plant material amount (A) ˃ Water volume (B) ˃ NaCl percent in water(C)). It is worthy to mention that the magnitude of effect of plant material amount was higher by 6 folds compared to NaCl percent in water. Figure 4b shows that the plant material had the highest increasing effect on the amount of citronellol compared to water volume, while the NaCl percent in water gave non-significant effect.

Figure 4c displays the increasing effect of the water volume on the amount of gamma-Eudesmol, which was higher than the NaCl increasing effect, while the plant material had a decreasing effect.

On the other hand, the only significant effects in case of citronellol acetate and rose oxide were the increasing effect of water volume on the amount of citronellol acetate Fig. 4d and the increasing effect of plant material amount on the amount of rose oxide Fig. 4e. Other studied factors showed non-significant effects on the studied responses.

#### Multiple response optimization

A multiple response optimization approach was performed for the optimization run with the goal of maximized Y1 to give the highest yield. Importantly, the numerical optimization of desirability function is used to estimate the optimization levels for each extraction run, Table 5^8,43^. In the Design-Expert, “the numerical optimization tool is usually based on the desirability function of selected responses and factors”. The desirability is based on “a mathematical method to find the optimum and transform the response variable to a 0–1 scale”^44^. The desirability of 1 represents the most desirable response and 0 represents a completely undesirable response^45^.

**Table 5:** 95% confidence interval.

Response	Goal	Actual	Prediction	SD	SE(n = 1)	Confidence = 90%		Confidence = 95%	
						90% PI low	90% PI high	95% PI low	95% PI high
Yield (Y1)	Maximize	0.4	0.385779	0.0250565	0.0288756	0.334643	0.436916	0.323397	0.448161
Citronellol (Y2)	Is in range	37.21	46.7289	1.49996	1.92978	43.2313	50.2265	42.4291	51.0287
Geraniol (Y3)	Is in range	6.03	8.63006	2.94209	3.78516	1.76961	15.4905	0.196208	17.0639
Gamma-Eudesmil (Y4)	Is in range	8.63	10.1622	0.521019	0.701584	8.83299	11.4914	8.50322	11.8212
Citronellol acetate (Y_5_)	Is in range	8.6	9.9405	0.655007	0.754844	8.60372	11.2773	8.30976	11.5712
I-Menthone (Y_6_)	Is in range	5.77	6.27941	0.412893	0.424864	5.53765	7.02117	5.37874	7.18008
Linalyl acetate (Y_7_)	Is in range	2.84	2.50744	0.41975	0.540031	1.52866	3.48623	1.30418	3.7107
Rose oxide (Y_8_)	Is in range	1.76	2.01716	0.220096	0.283166	1.50393	2.53039	1.38623	2.64809

**Figure 5:**
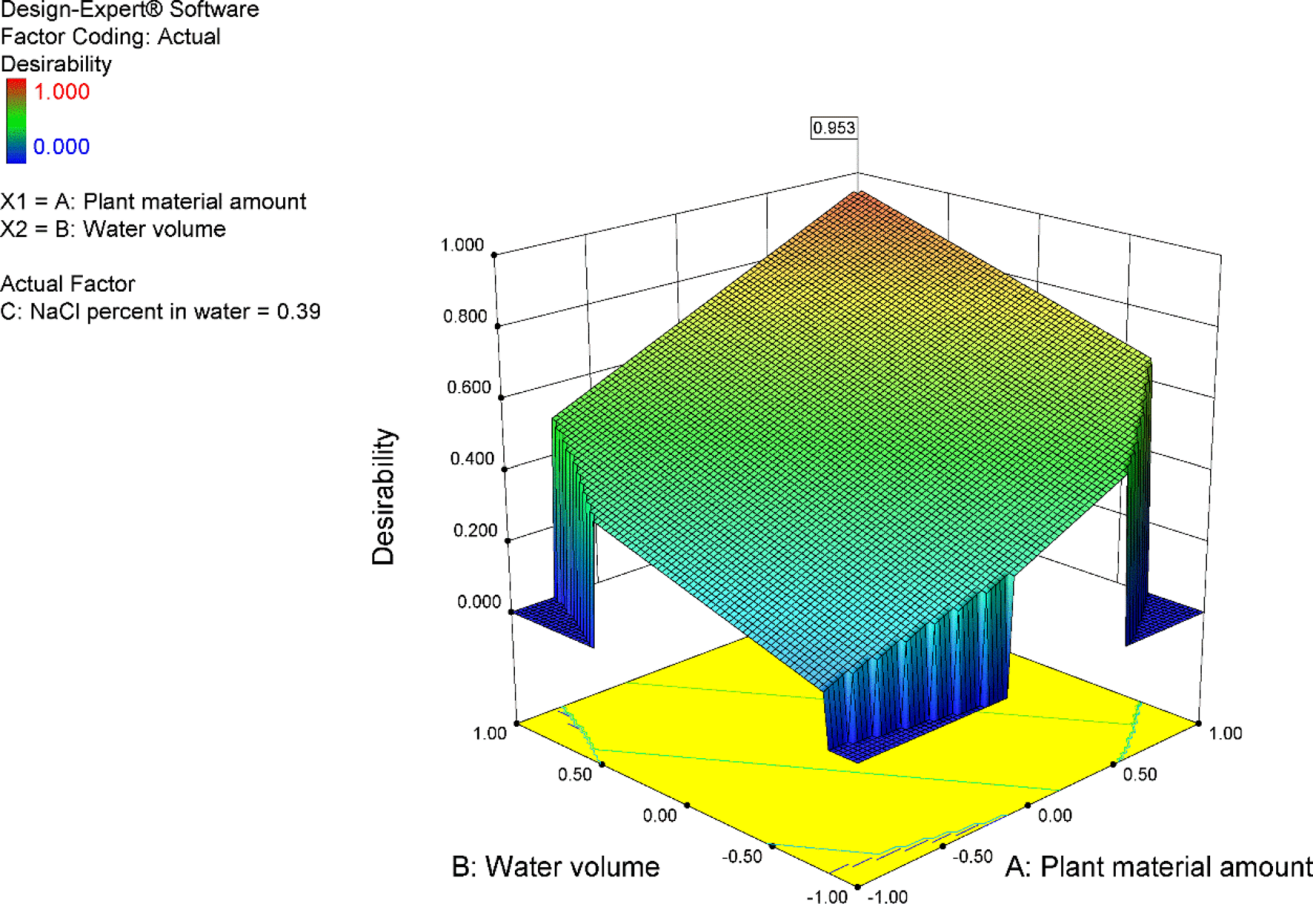
Desirability 3D plots for the optimized response (Yield Y1).

The numerical optimization run was based on increasing the yield (Y1) to maximum by putting the 3-factors (A, B and C) and the other responses in range with the desirability of 0.953 as shown in Fig. 5.

Table 6 demonstrates that the actual yield of the oil (0.4 ml) was in the range of 95% confidence interval (from 0.323397 to 0.448161). So, it confirmed the sufficiency of the response model as it established the expected optimization. Therefore, the model is accurate, satisfactory and the Box-Behnken design could be successfully used for the optimization of the amount of the yield of *P. graveolens* essential oil.

**Table 6:** Antimicrobial activity of the essential oil.

No	Strains	Zone of inhibition mm	MIC µl/mL
1	MRSA DFN1	13.8 ± 0.74	40.0 ± 1.54
2	MRSA DFN2	13.6 ± 1.4	40.0 ± 0.52
3	MSSA	15.3 ± 0.46	8.0 ± 1.05
4	*S. mutans*	11.7 ± 0.52	40.0 ± 2.3
5	*P. aeruginosa*	16.8 ± 1.3	20.0 ± 1.66
6	*K. pneumonia*	18.2 ± 1.9	1.6 ± 0.44
7	*C. albicans*	15.9 ± 0.25	40.0 ± 2.0

### Docking studies

The reported pharmacophoric features of UPPSIs are lipophilic tail and pyrophosphate mimetic “hydrogen bond donor and/or hydrogen bond acceptor" moieties^16^. From the medicinal point of view, it was observed that citronellol has the pharmacophoric features of UPPSIs (Supplementary Fig. 1). So that, citronellol may exert a synergistic antimicrobial activity with β-lactam antibiotics. To confirm the possible effect of citronellol as UPPSI, we carried out in silico model (flexible alignment and docking studies). The in vitro studies were performed to investigate the possible synergistic antimicrobial activity of citronellol with different β-lactam antibiotics against β-lactam resistant bacteria.

3D- flexible alignment of citronellol with Farnesyl diphosphate was presented in Supplementary Fig. 2. In general, the structure of citronellol is superimposed with the active site of the reference ligand (Farnesyl diphosphate). Additionally, citronellol indicated the same spatial orientation of Farnesyl diphosphate.

Undecaprenyl pyrophosphate synthase (UPPS) is considered as a key enzyme in the biosynthesis of cell wall of bacteria and represents an important target for the design of antibacterial drugs. Studying of the pharmacophoric features of UPPSIs revealed that citronellol has the same features of UPPSIs. Supplementary Fig. 2, indicates that the 3D- flexible alignment of citronellol has high structural similarity with Farnesyl diphosphate. Accordingly, it can be deduced that citronellol can serve as a promising inhibitor for UPPS. So that, docking studies were carried out to predict the binding mode of citronellol with its prospective biological target (UPPS).

Using Molecular Operating Environment (MOE, 2019), the active compounds were docked against undecaprenyl pyrophosphate synthase (PDB ID: 1V7U, resolution: 2.35 Å) in order to explain the produced antibacterial activity at molecular level. Farnesyl diphosphate was used as a reference molecule. Interestingly, the binding mode and the binding energy were an important criterion to evaluate the efficiency of binding against the active site.

Binding free energies (∆G in Kcal/mol) of β Citronellol against undecaprenyl pyrophosphate synthase compared to the reference molecule (Farnesyl diphosphate). Citronellol = − 75.5682678 kcal/mol , while Farnesyl diposhphate = − 169.371094 kcal/mol.

#### Validation

To validate the docking process, docking procedure was performed for the co-crystallized ligand (Farnesyl diphosphate) in the active site of undecaprenyl pyrophosphate synthase. The small RMSD value between the docked pose and the co-crystallized ligand showed the good feasibility of the applied methodology for the intended docking experiments (1.8 Å).

The co-crystalized ligand (Farnesyl diphosphate) indicated a binding sore of − 169.371094 kcal/mol against undecaprenyl pyrophosphate synthase. The diphosphate moiety was fitted into the hydrophilic pocket of the active site forming 9-hydrogen bonds with Arg194, Arg30, Gly29, Asn28, Gly27, Arg39, Arg77. In addition, it formed four electrostatic attractions with Arg77 and Asp26. The aliphatic lipophilic tail was oriented into the second pocket forming 7-hydrophobic bonds with Met25, Leu85, Val50, Ala69, and Leu88.

**Figure 6:**
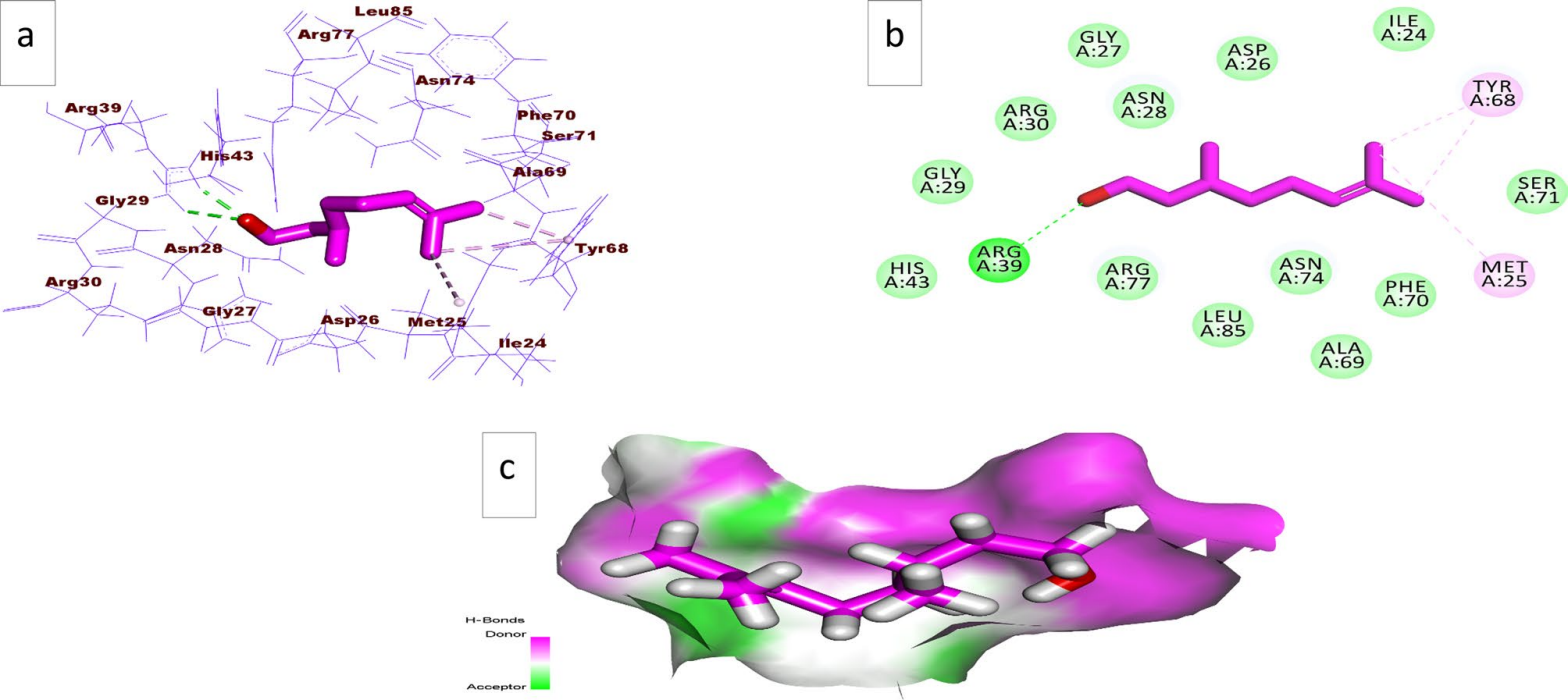
(**a**) 3D of Citronellol docked into the active site of undecaprenyl pyrophosphate synthase. (**b**) 2D of Citronellol docked into the active site of undecaprenyl pyrophosphate synthase, c) Surface map of Citronellol docked into the active site of undecaprenyl pyrophosphate synthase.

Citronellol demonstrated a binding affinity of − 75.5682678 kcal/mol against undecaprenyl pyrophosphate synthase. It showed a correct binding mode and orientation in the active site, Fig. 6. The hydroxyl moiety was oriented into the hydrophilic pocket of the active site forming 1-hydrogen bond with Arg39. Importantly, the aliphatic lipophilic tail was oriented into the second pocket forming 3-hydrophobic bonds with Met25 and Tyr68.

### Biological results

#### Antimicrobial and MIC

The optimized oil exhibited antimicrobial action against both *Gram*-positive and *Gram*-negative bacteria, as well as yeast *C. albicans*. This extract inhibited the growth of *Gram-*negative “*K. pneumonia* and *P. aeruginosa* ” bacteria, more than *Gram* positive “*S. aureus* and *S. mutans*”, as measured by the diameter of the inhibition zone in the agar plate. In the case of Gram-negative *K. pneumonia* with MIC 1.6 ± 0.44 µl/mL, the inhibition zone measured 18.2 ± 1.9 mm in diameter. The lowest impact of the oil extract against *S. mutans* was 11.7 ± 0.52 mm inhibitory zone diameter with MIC 40.0 ± 2.3 µl/mL.

#### Drug combination

Combination therapy has been demonstrated to be a promising technique for treating severe microbial infections. According to studies, combining multiple types of therapeutically antibacterial medications could increase bactericidal power, reduce, or eliminate adverse effects, and even overcome the multiple drug resistance via distinct mechanisms of action. Thus, in this investigation, the optimized oil was combined with various antibiotics (Oxacillin, Cloxacillin, Cefoxitin, and Vancomycin) against MRSA clinical strains. The optimized oil exerted a synergistic effect with Cefoxitin, Cloxacillin, Oxacillin and Vancomycin in both clinical isolates (DNF1 and DNF2) on the agar plates by increasing the zone of inhibition (Table 7, Supplementary Fig. 5). The fractional inhibitory concentration index (FIC) was determined, and the results exhibited presence of synergistic effect (FIC ≤ 0.5) with the tree antibiotics (Table 8) which revealed that this oil did not compete with these antibiotics for binding the target pathogen.

**Table 7:** Drug combination activity of the essential oil on agar plates.

	Zone of inhibition mm			
	Antibiotic		Extract	
	Alone	Combined	Alone	Combined
MRSA DFN1				
Cefoxitin	16.6 ± 0.25	31.2 ± 1.25	13.8 ± 0.74	31.2 ± 1.25
Cloxacillin	16.2 ± 0.66	24.2 ± 0.95		24.2 ± 0.95
Oxacillin	0.0 ± 0.0	0.0 ± 0.0		24.3 ± 0.49
Vancomycin	17.2 ± 0.64	32.3 ± 2.1		32.3 ± 0.46
MRSA DFN2				
Cefoxitin	0.0 ± 0.0	15.4 ± 0.36	13.6 ± 1.4	15.4 ± 0.36
Cloxacillin	0.0 ± 0.0	16.8 ± 1.1		16.8 ± 1.1
Oxacillin	0.0 ± 0.0	17.4 ± 0.61		17.4 ± 0.61
Vancomycin	18.2 ± 1.46	28.8 ± 0.94		28.8 ± 0.94

**Table 8:** FIC index for the combination effects of the essential oil with different antibiotics against MRSA.

Antibiotic ^(1)^	MIC for MRSA DFN1				MIC for MRSA DFN2			
	Alone		Combined		Alone		Combined	
	E µl/mL	A µg/mL	E µl/mL	A µg/mL	E µl/mL	A µg/mL	E µl/mL	A µg/mL
Cefoxitin	40	8.0	10	2.0	40	16	5	2.0
Cloxacillin	40	5.0	10	1.0	40	8.0	10	2.0
Oxacillin	40	10	10	2.0	40	10	5	1.0
Vancomycin	40	2.0	10	0.5	40	2.0	10	0.5

	FIC index							
	Value	Effect	Value	Effect				
Cefoxitin	0.5	Synergistic	0.25	Synergistic				
Cloxacillin	0.45	Synergistic	0.5	Synergistic				
Oxacillin	0.45	Synergistic	0.225	Synergistic				
Vancomycin	0.5	Synergistic	0.5	Synergistic				

MIC: Minimum inhibitory concentration; mg/ml milligram /milliliter.

**E**: Extract.

**A**: Antibiotic.

The fractional inhibitory concentration index (FIC) = (MIC of extract E combined/MIC of extract E alone) + ‏ (MIC of Antibiotic A combined/MIC of Antibiotic A alone). FIC index ≤ 1, synergistic effect; 1 < FIC index ≤ 2, additive effect; FIC index > 2, antagonistic effect.

#### Biofilm inhibition activity

**Figure 7:**
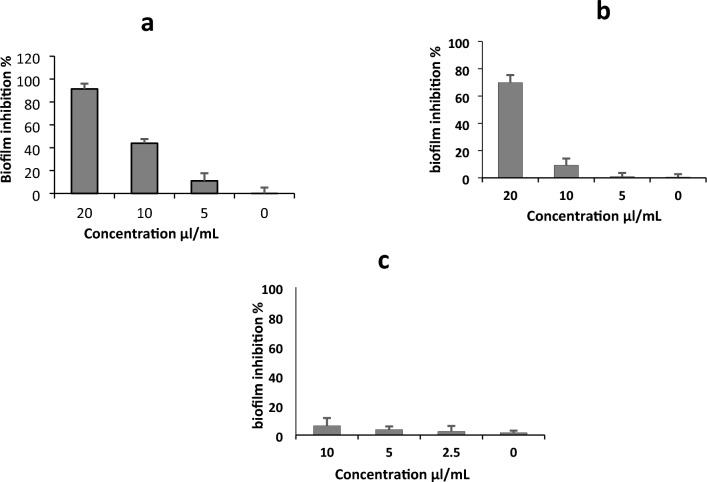
Biofilm inhibition percentage of the essential oil against the biofilm fotmation of (**a**) *S. aureus* (MRSA) strain, (**b**) C. *albicans*, (**c**) *P. aeruginosa*. The figure illustrates the effect of different doses of the essential oil under the MIC value on the biofilm formation.

At concentrations lower than the lethal dose (20, 10, and 5 µl/ml), the essential oil showed remarkable antibiofilm action (*P* < 0.05) against *S. aureus* and *C. albicans*. These concentrations didn't affect the bacterial cell growth as MIC determined previously as shown in Table 6. The oil was exhibited significant inhibition of biofilm up to 91.4% (*P* < 0.01) at concentration 20 µl/mL against *S. aureus* highly biofilm producing strain in dose dependent manner where the biofilm inhibition proportion was reduced to 43.9 and 11.07% at concentrations 10 and 5 µl/mL respectively as shown in Fig. 7a. Also, *C. albicans* biofilm formation was affected by the optimized oil at concentration 20 µl/mL where the reduction proportion was reached to 69.81% (*P* < 0.05) as shown in Fig. 7b. On the other hand, *P. aeruginosa* biofilm formation wasn't affected by the sub lethal dose of the oil as shown in Fig. 7c.

## Conclusion

This study provides a comprehensive GC–MS profiling of Egyptian *Pelargonium graveolens* oil at the four different seasons. Autumn showed the highest yield of the oil and hence used for the optimization study. Importantly, the optimization of the essential oil of *P. graveolens* using (3^3^) Box-Behnken design was proved to be a successful method to determine the optimized run with the highest yield of the oil. 3-factors that significantly affect the quantity of the yield were studied (plant material amount, water volume and NaCl percent in water). Moreover, the study revealed that increasing the plant material increased the yield of the oil by 1.5 folds compared to water volume. Despite the significant effect of NaCl percent in water on the yield of oil but, it had the least effect among the studied factors (it gave only one-sixth the effect of plant material amount). Numerical optimization was successfully carried out with the actual yield of the optimized oil was in the range of 95% confidence interval.

Docking results showed high binding affinity of citronellol towards UPPS synthase with synergistic antimicrobial activity with different *β*-lactam antibiotic against resistant bacteria. Based on MIC and biofilm inhibition activity study, the optimized essential oil demonstrated a good antimicrobial activity against MRSA strains. This study could be the nucleus for further studies on* P. graveolens.*

### Supplementary Information

Supplementary Information 1.

Supplementary Information 2.

## Data Availability

The datasets presented in the current study are available from the corresponding author on reasonable request.
