# Supplementary material for: Optimization of oil yield of Pelargonium graveolens L'Hér using Box-Behnken design in relation to its antimicrobial activity and in silico study
Source: Sci Rep. 2023 Nov 14;13:19887. doi: 10.1038/s41598-023-47170-0 (PMC10645939; doi:10.1038/s41598-023-47170-0)

**Supplementary figure legends:**

Supplementary Figure 1: Structural pharmacophoric features of UPPS native substrate farnesyl pyrophosphate (FPP), and Undecaprenyl Pyrophosphate Synthase Inhibitors (UPPSIs).

Supplementary Figure 2: Citronellol has the same pharmacophoric features of Farnesyl diphosphate.

Supplementary Figure 3: Superimposition of the co-crystallized ligand (Farnesyl diphosphate) of undecaprenyl pyrophosphate synthase (carbon atoms in green) and the docked pose of the same ligand (carbon atoms in pink).

Supplementary Figure 4: a) 3D of the co-crystallized ligand (Farnesyl diphosphate) docked into the active site of undecaprenyl pyrophosphate synthase. b) 2D of Farnesyl diphosphate docked into the active site of undecaprenyl pyrophosphate synthase, c) Surface map of Farnesyl diphosphate docked into the active site of undecaprenyl pyrophosphate synthase.

Supplementary Figure 5: Drug combination test using agar well and disc diffusion assay.

Supplementary Figure 1: Structural pharmacophoric features of UPPS native substrate farnesyl pyrophosphate (FPP), and Undecaprenyl Pyrophosphate Synthase Inhibitors (UPPSIs).

Supplementary Figure 2: Citronellol (carbon atoms in blue) has the same pharmacophoric features of Farnesyl diphosphate (carbon atoms in green).

Supplementary Figure 3: Superimposition of the co-crystallized ligand (Farnesyl diphosphate) of undecaprenyl pyrophosphate synthase (carbon atoms in green) and the docked pose of the same ligand (carbon atoms in pink).


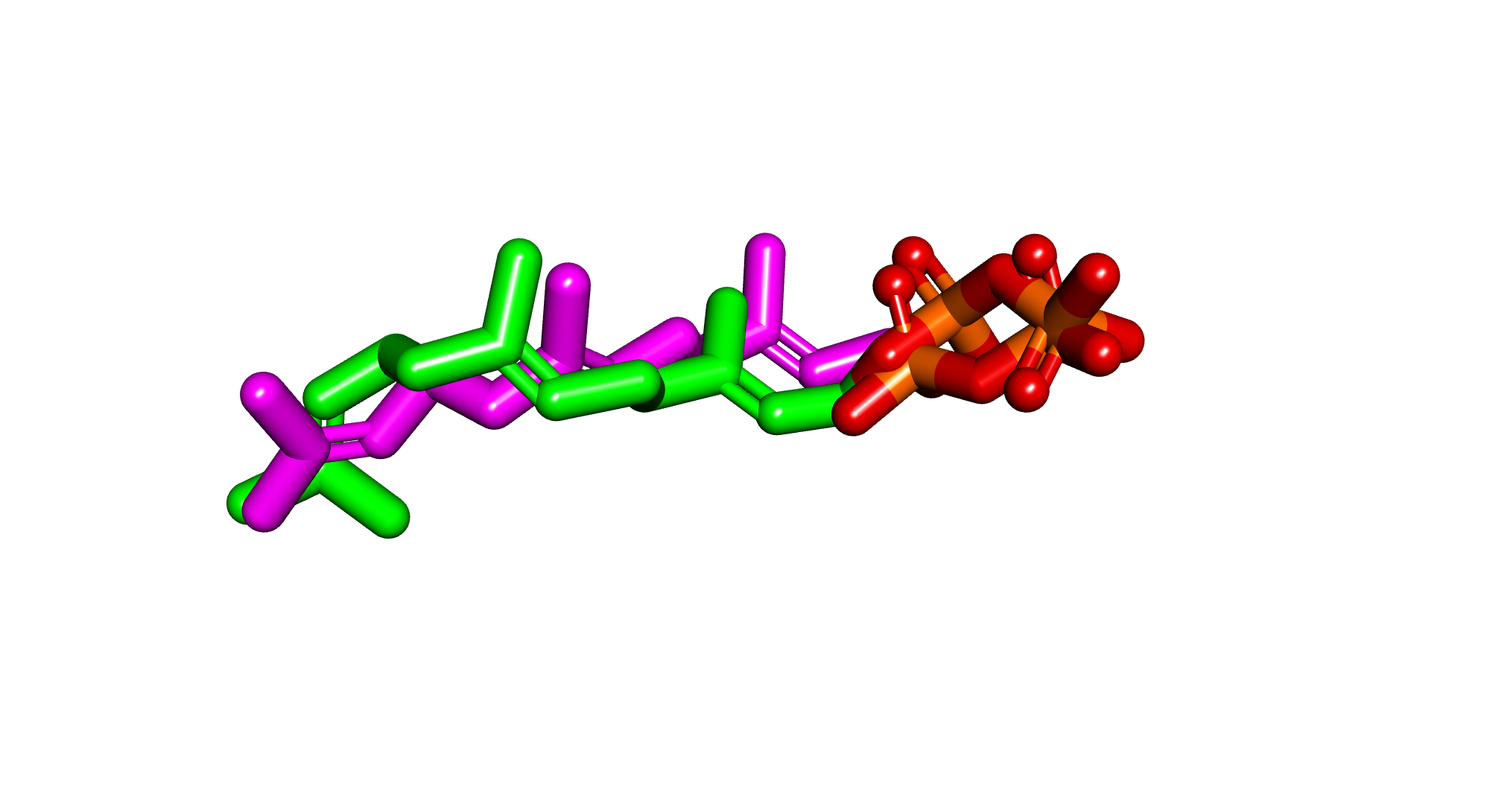


Supplementary Figure 4: a)3D of the co-crystallized ligand (Farnesyl diphosphate) docked into the active site of undecaprenyl pyrophosphate synthase. b) 2D of Farnesyl diphosphate docked into the active site of undecaprenyl pyrophosphate synthase, c) Surface map of Farnesyl diphosphate docked into the active site of undecaprenyl pyrophosphate synthase.


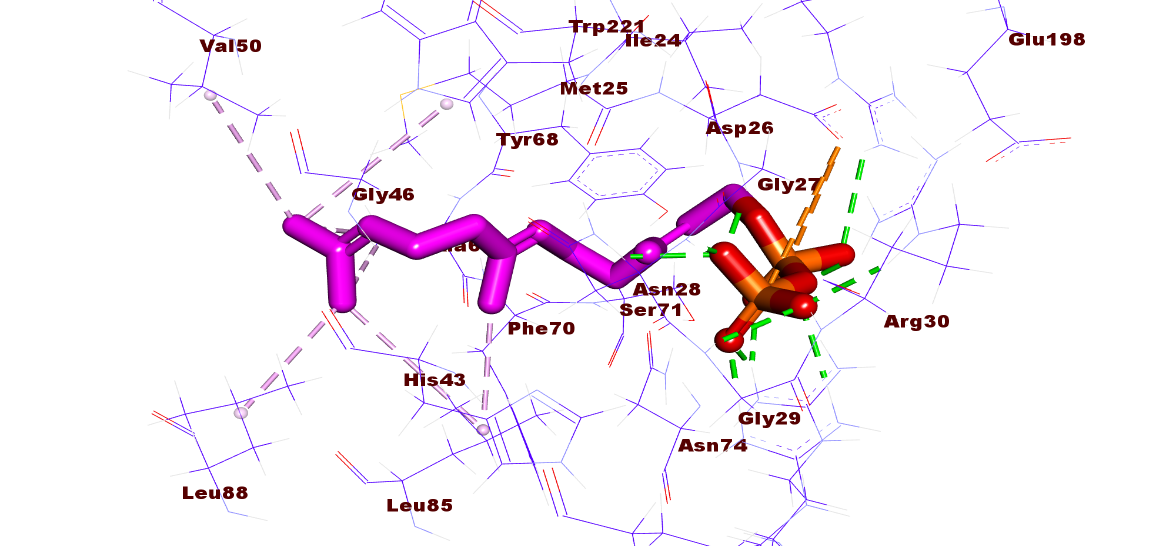

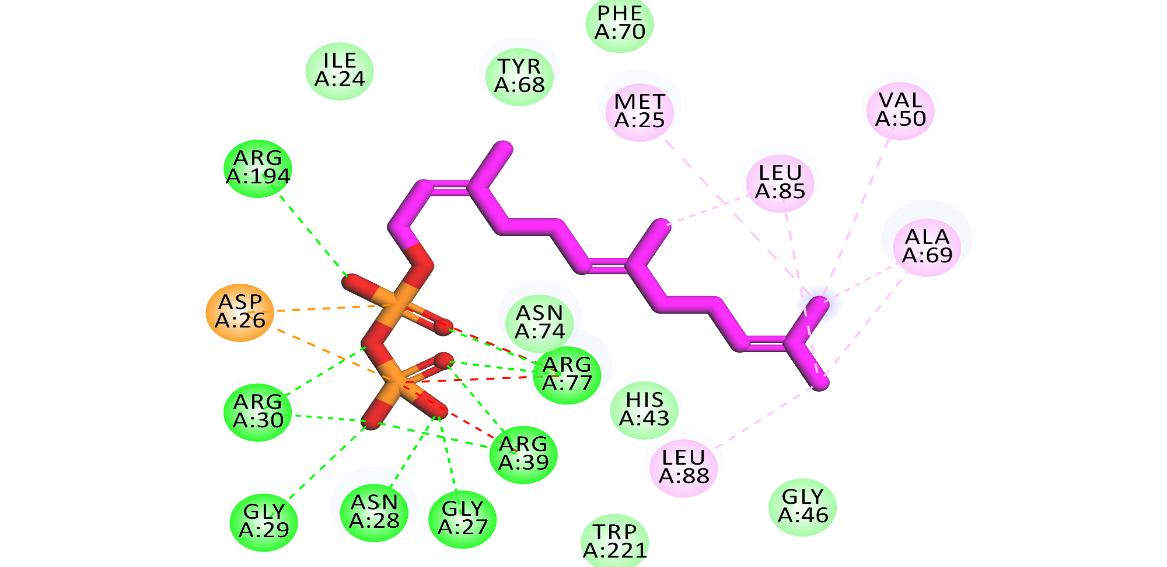

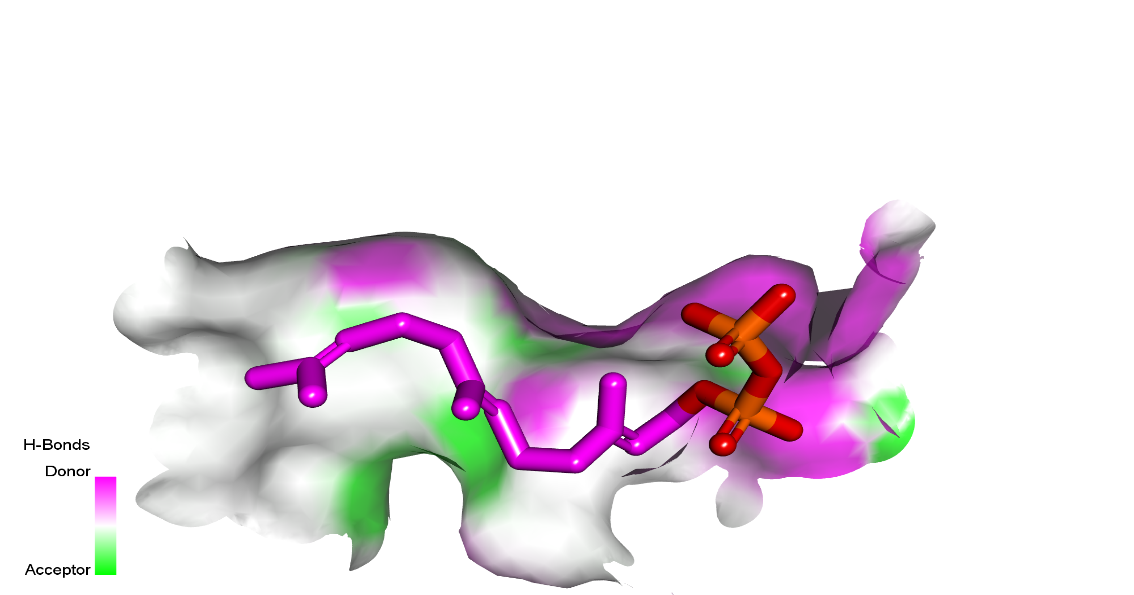


**b**

**a**

**c**

Supplementary Figure 5: Drug combination test using agar well and disc diffusion assay.


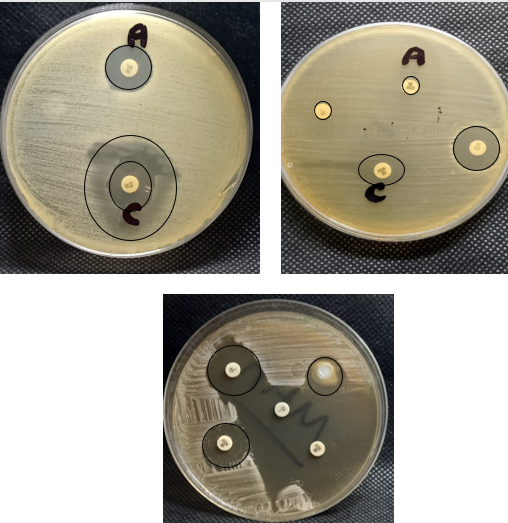

Supplement: Supplementary file 1 — Supplementary Information 1. [file 41598_2023_47170_MOESM1_ESM.docx]
