# Supplementary material for: Optimization of oil yield of Pelargonium graveolens L'Hér using Box-Behnken design in relation to its antimicrobial activity and in silico study
Source: Sci Rep. 2023 Nov 14;13:19887. doi: 10.1038/s41598-023-47170-0 (PMC10645939; doi:10.1038/s41598-023-47170-0)
Supplement: Supplementary file 2 — Supplementary Information 2. [file 41598_2023_47170_MOESM2_ESM.docx]

**Supplementary table legends:**

Supplementary Table 1: Comparison between different seasons GC-MS analysis in the quantity of the active constituents and yield of the oil.

Supplementary Table 2

Supp. Table 2(a): Sequential model fitting for the yield of oil (Y1). *Statistically significant: p˂0.05.

Supp. Table 2(b): Sequential model fitting for the Citronellol(Y2). *Statistically significant: p˂0.05.

Supp. Table 2(c): Sequential model fitting for the Geraniol(Y3). *Statistically significant: p˂0.05.

Supp. Table 2(d): Sequential model fitting for the gamma-Eudesmol(Y4). *Statistically significant: p˂0.05.

Supp. Table 2(e): Sequential model fitting for the Citronellol acetate(Y5). *Statistically significant: p˂0.05.

Supp. Table 2(f): Sequential model fitting for the I- Menthone(Y6). *Statistically significant: p˂0.05.

Supp. Table 2(g): Sequential model fitting for the Linalyl acetate(Y7). *Statistically significant: p˂0.05.

Supp. Table 2(h): Sequential model fitting for the Rose oxide(Y8). *Statistically significant: p˂0.05.

Supplementary Table 1: Comparison between different seasons GC-MS analysis in the quantity of the active constituents and yield of the oil.

| Compound | Summer (Area%) | Winter (Area%) | Spring (Area%) | Autumn (Area%) | Kovates index (K_i_) |
| --- | --- | --- | --- | --- | --- |
| Monocyclic Monoterpenes | | | | | |
| Rose oxide | 7.87 | --------- | 8.18 | 3.96 | 1114 |
| Limonen | 0.35 | 0.77 | 0.38 | ------------ | 1407 |
| I-Menthone | 11.90 | 11.65 | 11.18 | 5.13 | 1148 |
| Levomenthol | 0.61 | 0.21 | 0.63 | 0.70 | 1164 |
| Bicyclic Monoterpenes | | | | | |
| à-Pinene | 0.65 | 0.78 | 0.68 | ---------- | 1542 |
| β-Pinene | --------- | 0.63 | ---------- | --------- | 1580 |
| Acyclic Monoterpenes | | | | | |
| Linalool | --------- | 7.98 | -------- | 0.71 | 1082 |
| Linalyl acetate | 1.53 | 1.23 | 1.33 | 3.03 | 1272 |
| Citronellol | 12.96 | 10.28 | 8.39 | 33.01 | 1179 |
| Geraniol | 3.57 | 8.16 | 5.37 | 1.33 | 1228 |
| Linalool, formate | 0.32 | --------- | --------- | ------------ | 1270 |
| Sesquiterpenes | | | | | |
| à-Cubebene | 0.17 | ------- | 0.27 | --------- | 1344 |
| alpha-Copaene | 0.53 | 0.67 | 1.02 | ------- | 1377 |
| à-Bourbonene | 3.27 | 3.21 | 3.69 | 0.47 | 1344 |
| Caryophyllene | 1.81 | 1.80 | 2.42 | 0.89 | 1494 |
| Germacra-1(10),4(15),5-Triene | ------------ | ---------- | 0.12 | -------- | 1660 |
| Cadina-1(10),4-diene | ----- | ------- | ----------- | 1.66 | 1580 |
| Humulene | 0.35 | ------- | 0.56 | ------- | 1461 |
| Germacrene D | 2.15 | 3.49 | 2.89 | 2.15 | 1515 |
| Aromandendrene | --------- | 3.72 | 4.98 | -------- | 1281 |
| Alloaromadendrene | 0.42 | ------- | ---------- | --------- | 1288 |
| Cubebol | 0.88 | 1.38 | 0.94 | --------- | 1484 |
| à-Cadinene | 5.19 | 4.32 | 6.33 | ------- | 1221 |
| à-Guaiene | -------- | 0.15 | 0.22 | ------- | 1523 |
| Spathulenol | --------- | ----------- | --------- | 0.51 | 1146 |
| Caryophyllene oxide | ---------- | ---------- | ---------- | 0.69 | 1507 |
| Cis-Lanceol | 4.60 | ----------- | --------- | ------------- | 1060 |
| Aromadendrene oxide | ----------- | ------------ | 4.09 | -------------- | 1506 |
| δ - Eudesmol | 14.64 | 15.34 | 13.72 | 17.28 | 1626 |
| Cubenol | 2.05 | 0.86 | 2.47 | 2.6 | 1440 |
| Agarospirol | ------------ | ----------- | --------- | 0.34 | 1598 |
| à-Eudesmol | 3.81 | 2.78 | 3.60 | --------- | 1398 |
| Farnesol | 0.61 | 0.17 | 1.01 | ------------ | 1710 |
| Fatty alcohol esters | | | | | |
| Citronellyl Formate | 1.91 | -------- | 1.21 | 13.22 | 1601 |
| Geranyl acetate | 4.48 | 1.59 | 2.30 | --------- | 1352 |
| Geranyl Formate | --------- | 0.84 | ------- | 0.70 | 1501 |
| Citronellol acetate | 1.43 | 0.58 | 1.41 | 1.36 | 1302 |
| Citronellyl propionate | 3.56 | ----------- | ------- | 2.18 | 1402 |
| Citronellyl butyrate | ---------- | --------- | ---- | 2.65 | 1437 |
| Geranyl propionate | 2.75 | 2.66 | 2.65 | ------- | 1550 |
| Citronellyl tiglate | ----------- | ------------- | ----------- | 1.04 | 1586 |
| Geranyl tiglate | 2.93 | 5.03 | 3.79 | 0.74 | 1635 |
| Geranyl heptanoate | ------------ | 0.22 | 0.28 | ------------ | 1542 |
| Carboxylic acid esters |  |  |  |  |  |
| Butanoic acid, 3,7-Dimethyl-2,6-octadienyl ester | 4.07 | 4.67 | 4.42 | 2.07 | 1536 |
| Hexanoic acid,3,7-dimethyl-2,6-octadienyl ester | --------- | 1.43 | 0.46 | -------------- | 1700 |
| Yield of essential oil in ml / Kg | 0.8 ml | 1 ml | 1 ml | 1.6 ml |  |
| Total area % in each season |  |  |  |  |  |
| Monocyclic Monoterpenes | 20.73 | 12.63 | 20.37 | 9.79 |  |
| Bicyclic Monoterpenes | 0.65 | 1.41 | 0.68 | --------- |  |
| Acyclic Monoterpenes | 18.38 | 27.65 | 15.09 | 38.08 |  |
| Sesquiterpenes | 40.48 | 37.89 | 48.33 | 26.59 |  |
| Fatty alcohol esters | 17.06 | 10.92 | 11.64 | 21.89 |  |
| Carboxylic acid esters | 4.07 | 6.1 | 4.88 | 2.07 |  |

Supplementary Table 2

Supp. Table 2(a): Sequential model fitting for the yield of oil (Y1). *Statistically significant: p˂0.05.

| Source | Sum of squares | Mean square | DF | F-value | P-value | Remarks |
| --- | --- | --- | --- | --- | --- | --- |
| Sequential sum of squares | | | | | | |
| Mean | 1.11 | 1.11 | 1 |  |  |  |
| Linear | 0.066 | 0.022 | 3 | 35.17 | ˂0.0001* | Suggested |
| 2FI | 1.875E-003 | 6.250E-004 | 3 | 0.99 | 0.4347 |  |
| Quadratic | 1.787E-003 | 5.956E-004 | 3 | 0.93 | 0.4764 |  |
| Cubic | 2.500E-003 | 8.333E-004 | 3 | 1.67 | 0.3099 | Aliased |
| Residual | 2.000E-003 | 5.000E-004 | 4 |  |  |  |
| Total | 1.19 | 0.070 | 17 |  |  |  |
| Lack of fit tests | | | | | | |
| Linear | 6.162E-003 | 6.846E-004 | 9 | 1.37 | 0.4065 | Suggested |
| 2FI | 4.287E-003 | 7.145E-004 | 6 | 1.43 | 0.3804 |  |
| Quadratic | 2.500E-003 | 8.333E-004 | 3 | 1.67 | 0.3099 |  |
| Cubic | 0.000 |  | 0 |  |  | Alaised |
| Pure error | 2.000E-003 | 5.000E-004 | 4 |  |  |  |
|  | **Std.Dev** | **R-Squared** | **Adjusted**  **R-Squared** | **Predicted**  **R-Squared** | **PRESS** |  |
| Model summary statistics source | | | | | | |
| Linear | 0.025 | 0.8903 | 0.8650 | 0.7974 | 0.015 | Suggested |
| 2FI | 0.025 | 0.9155 | 0.8648 | 0.6782 | 0.024 |  |
| Quadratic | 0.025 | 0.9395 | 0.8618 | 0.4205 | 0.043 |  |
| Cubic | 0.022 | 0.9731 | 0.8925 |  | + | Aliased |

Supp. Table 2(b): Sequential model fitting for the Citronellol(Y2). *Statistically significant: p˂0.05.

| Source | Sum of squares | Mean square | DF | F-value | P-value | Remarks |
| --- | --- | --- | --- | --- | --- | --- |
| Sequential sum of squares | | | | | | |
| Mean | 24986.18 | 24986.18 | 1 |  |  |  |
| Linear | 126.92 | 42.31 | 3 | 11.65 | 0.0005 |  |
| 2FI | 24.69 | 8.23 | 3 | 3.66 | 0.0517 | Suggested |
| Quadratic | 4.66 | 1.55 | 3 | 0.61 | 0.6297 |  |
| Cubic | 16.64 | 5.55 | 3 | 18.56 | 0.0082 | Aliased |
| Residual | 1.20 | 0.30 | 4 |  |  |  |
| Total | 25160.29 | 1480.02 | 17 |  |  |  |
| Lack of fit tests | | | | | | |
| Linear | 46.00 | 5.11 | 9 | 17.10 | 0.0075 |  |
| 2FI | 21.30 | 3.55 | 6 | 11.88 | 0.0158 | Suggested |
| Quadratic | 16.64 | 5.55 | 3 | 18.56 | 0.0082 |  |
| Cubic | 0.000 |  | 0 |  |  | Aliased |
| Pure error | 1.20 | 0.30 | 4 |  |  |  |
|  | **Std.Dev** | **R-Squared** | **Adjusted**  **R-Squared** | **Predicted**  **R-Squared** | **PRESS** |  |
| Model summary statistics source | | | | | | |
| Linear | 1.91 | 0.7290 | 0.6664 | 0.4414 | 97.26 |  |
| 2FI | 1.50 | 0.8708 | 0.7932 | 0.3724 | 109.28 | Suggested |
| Quadratic | 1.60 | 0.8976 | 0.7658 | -0.5400 | 268.13 |  |
| Cubic | 0.55 | 0.9931 | 0.9725 |  | + | Aliased |

Supp. Table 2(c): Sequential model fitting for the Geraniol(Y3). *Statistically significant: p˂0.05.

| Source | Sum of squares | Mean square | DF | F-value | P-value | Remarks |
| --- | --- | --- | --- | --- | --- | --- |
| Sequential sum of squares | | | | | | |
| Mean | 2955.75 | 2955.75 | 1 |  |  |  |
| Linear | 69.15 | 23.05 | 3 | 1.78 | 0.2003 |  |
| 2FI | 81.65 | 27.22 | 3 | 3.14 | 0.0737 | Suggested |
| Quadratic | 32.93 | 10.98 | 3 | 1.43 | 0.3121 |  |
| Cubic | 1.49 | 0.50 | 3 | 0.038 | 0.9886 | Aliased |
| Residual | 52.14 | 13.03 | 4 |  |  |  |
| Total | 3193.10 | 187.83 | 17 |  |  |  |
| Lack of fit tests | | | | | | |
| Linear | 116.07 | 12.90 | 9 | 0.99 | 0.5490 |  |
| 2FI | 34.42 | 5.74 | 6 | 0.44 | 0.8235 | Suggested |
| Quadratic | 1.49 | 0.50 | 3 | 0.038 | 0.9886 |  |
| Cubic | 0.000 |  | 0 |  |  | Aliased |
| Pure error | 52.14 | 13.03 | 4 |  |  |  |
|  | **Std.Dev** | **R-Squared** | **Adjusted**  **R-Squared** | **Predicted**  **R-Squared** | **PRESS** |  |
| Model summary statistics source | | | | | | |
| Linear | 3.60 | 0.2913 | 0.1278 | -0.2271 | 291.27 |  |
| 2FI | 2.94 | 0.6353 | 0.4165 | 0.1919 | 191.81 | Suggested |
| Quadratic | 2.77 | 0.7741 | 0.4836 | 0.5562 | 105.34 |  |
| Cubic | 3.61 | 0.7803 | 0.1214 |  | + | Aliased |

Supp. Table 2(d): Sequential model fitting for the gamma-Eudesmol(Y4). *Statistically significant: p˂0.05.

| Source | Sum of squares | Mean square | DF | F-value | P-value | Remarks |
| --- | --- | --- | --- | --- | --- | --- |
| Sequential sum of squares | | | | | | |
| Mean | 1831.65 | 1831.65 | 1 |  |  |  |
| Linear | 11.70 | 3.90 | 3 | 4.45 | 0.0233 |  |
| 2FI | 6.01 | 2.00 | 3 | 3.72 | 0.0496 |  |
| Quadratic | 3.49 | 1.16 | 3 | 4.28 | 0.0517 | Suggested |
| Cubic | 0.39 | 0.13 | 3 | 0.35 | 0.7942 | Aliased |
| Residual | 1.51 | 0.38 | 4 |  |  |  |
| Total | 1854.75 | 109.10 | 17 |  |  |  |
| Lack of fit tests | | | | | | |
| Linear | 9.89 | 1.10 | 9 | 2.92 | 0.1575 |  |
| 2FI | 3.88 | 0.65 | 6 | 1.72 | 0.3130 |  |
| Quadratic | 0.39 | 0.13 | 3 | 0.35 | 0.7942 | Suggested |
| Cubic | 0.000 | 0 |  |  |  | Aliased |
| Pure error | 1.51 | 0.38 | 4 |  |  |  |
|  | **Std.Dev** | **R-Squared** | **Adjusted**  **R-Squared** | **Predicted**  **R-Squared** | **PRESS** |  |
| Model summary statistics source | | | | | | |
| Linear | 0.94 | 0.5065 | 0.3926 | 0.0487 | 21.97 |  |
| 2FI | 0.73 | 0.7668 | 0.6269 | 0.1416 | 19.83 |  |
| Quadratic | 0.52 | 0.9177 | 0.8119 | 0.6259 | 8.64 | Suggested |
| Cubic | 0.61 | 0.9347 | 0.7389 |  | + | Aliased |

Supp. Table 2(e): Sequential model fitting for the Citronellol acetate(Y5). *Statistically significant: p˂0.05.

| Source | Sum of squares | Mean square | DF | F-value | P-value | Remarks |
| --- | --- | --- | --- | --- | --- | --- |
| Sequential sum of squares | | | | | | |
| Mean | 1320.00 | 132.00 | 1 |  |  |  |
| Linear | 8.23 | 2.74 | 3 | 6.39 | 0.0068 | Suggested |
| 2FI | 0.42 | 0.14 | 3 | 0.27 | 0.8449 |  |
| Quadratic | 1.87 | 0.62 | 3 | 1.32 | 0.3410 |  |
| Cubic | 0.50 | 0.17 | 3 | 0.24 | 0.8666 | Aliased |
| Residual | 2.79 | 0.70 | 4 |  |  |  |
| Total | 1333.81 | 78.46 | 17 |  |  |  |
| Lack of fit tests | | | | | | |
| Linear | 2.78 | 0.31 | 9 | 0.44 | 0.8575 | Suggested |
| 2FI | 2.36 | 0.39 | 6 | 0.56 | 0.7474 |  |
| Quadratic | 0.50 | 0.17 | 3 | 0.24 | 0.8666 |  |
| Cubic | 0.000 |  | 0 |  |  | Alaised |
| Pure error | 2.79 | 0.70 | 4 |  |  |  |
|  | **Std.Dev** | **R-Squared** | **Adjusted**  **R-Squared** | **Predicted**  **R-Squared** | **PRESS** |  |
| Model summary statistics source | | | | | | |
| Linear | 0.66 | 0.5960 | 0.5028 | 0.3857 | 8.48 | Suggested |
| 2FI | 0.72 | 0.6264 | 0.4022 | 0.0423 | 13.22 |  |
| Quadratic | 0.69 | 0.7616 | 0.4550 | 0.1078 | 12.32 |  |
| Cubic | 0.84 | 0.7976 | 0.1903 |  | + | Aliased |

Supp. Table 2(f): Sequential model fitting for the I- Menthone(Y6). *Statistically significant: p˂0.05.

| Source | Sum of squares | Mean square | DF | F-value | P-value | Remarks |
| --- | --- | --- | --- | --- | --- | --- |
| Sequential sum of squares | | | | | | |
| Mean | 670.33 | 670.33 | 1 |  |  | Suggested |
| Linear | 0.36 | 0.12 | 3 | 0.66 | 0.5899 |  |
| 2FI | 0.59 | 0.20 | 3 | 1.11 | 0.3902 |  |
| Quadratic | 0.13 | 0.045 | 3 | 0.19 | 0.8996 |  |
| Cubic | 0.76 | 0.25 | 3 | 1.16 | 0.4287 | Aliased |
| Residual | 0.88 | 0.22 | 4 |  |  |  |
| Total | 673.05 | 39.59 | 17 |  |  |  |
| Lack of fit tests | | | | | | |
| Linear | 1.49 | 0.17 | 9 | 0.75 | 0.6689 |  |
| 2FI | 0.90 | 0.15 | 6 | 0.68 | 0.6796 |  |
| Quadratic | 0.76 | 0.25 | 3 | 1.16 | 0.4287 |  |
| Cubic | 0.000 | 0 |  |  |  | Aliased |
| Pure error | 0.88 | 0.22 | 4 |  |  |  |
|  | **Std.Dev** | **R-Squared** | **Adjusted**  **R-Squared** | **Predicted**  **R-Squared** | **PRESS** |  |
| Model summary statistics source | | | | | | |
| Linear | 0.43 | 0.1325 | -0.0676 | -0.4963 | 4.08 |  |
| 2FI | 0.42 | 0.3493 | -0.0412 | -1.0151 | 5.50 |  |
| Quadratic | 0.48 | 0.3984 | -0.3752 | -3.9777 | 13.58 |  |
| Cubic | 0.47 | 0.6780 | -0.2879 |  | + | Aliased |

Supp. Table 2(g): Sequential model fitting for the Linalyl acetate(Y7). *Statistically significant: p˂0.05.

| Source | Sum of squares | Mean square | DF | F-value | P-value | Remarks |
| --- | --- | --- | --- | --- | --- | --- |
| Sequential sum of squares | | | | | | |
| Mean | 164.61 | 164.61 | 1 |  |  | Suggested |
| Linear | 0.32 | 0.11 | 3 | 0.42 | 0.7398 |  |
| 2FI | 1.47 | 0.49 | 3 | 2.78 | 0.0961 | Suggested |
| Quadratic | 0.44 | 0.15 | 3 | 0.77 | 0.5443 |  |
| Cubic | 1.06 | 0.35 | 3 | 5.31 | 0.0702 | Aliased |
| Residual | 0.27 | 0.066 | 4 |  |  |  |
| Total | 168.16 | 9.89 | 17 |  |  |  |
| Lack of fit tests | | | | | | |
| Linear | 2.97 | 0.33 | 9 | 4.97 | 0.0687 |  |
| 2FI | 1.50 | 0.25 | 6 | 3.76 | 0.1103 | Suggested |
| Quadratic | 1.06 | 0.35 | 3 | 5.31 | 0.0702 |  |
| Cubic | 0.000 |  | 0 |  |  | Aliased |
| Pure error | 0.27 | 0.066 | 4 |  |  |  |
|  | **Std.Dev** | **R-Squared** | **Adjusted**  **R-Squared** | **Predicted**  **R-Squared** | **PRESS** |  |
| Model summary statistics source | | | | | | |
| Linear | 0.50 | 0.0889 | -0.1214 | -0.8350 | 6.51 |  |
| 2FI | 0.42 | 0.5033 | 0.2053 | -1.2519 | 7.99 | Suggested |
| Quadratic | 0.43 | 0.6271 | 0.1476 | -3.8869 | 17.34 |  |
| Cubic | 0.26 | 0.9252 | 0.7007 |  | + | Aliased |

Supp. Table 2(h): Sequential model fitting for the Rose oxide(Y8). *Statistically significant: p˂0.05.

| Source | Sum of squares | Mean square | DF | F-value | P-value | Remarks |
| --- | --- | --- | --- | --- | --- | --- |
| Sequential sum of squares | | | | | | |
| Mean | 34.99 | 34.99 | 1 |  |  |  |
| Linear | 0.47 | 0.16 | 3 | 1.68 | 0.2200 |  |
| 2FI | 0.74 | 0.25 | 3 | 5.08 | 0.0216 | Suggested |
| Quadratic | 0.094 | 0.031 | 3 | 0.56 | 0.6592 |  |
| Cubic | 0.15 | 0.050 | 3 | 0.84 | 0.5397 | Aliased |
| Residual | 0.24 | 0.060 | 4 |  |  |  |
| Total | 36.69 | 2.16 | 17 |  |  |  |
| Lack of fit tests | | | | | | |
| Linear | 0.98 | 0.11 | 9 | 1.82 | 0.2956 |  |
| 2FI | 0.24 | 0.041 | 6 | 0.68 | 0.6810 | Suggested |
| Quadratic | 0.15 | 0.050 | 3 | 0.84 | 0.5397 |  |
| Cubic | 0.000 |  | 0 |  |  | Aliased |
| Pure error | 0.24 | 0.060 | 4 |  |  |  |
|  | **Std.Dev** | **R-Squared** | **Adjusted**  **R-Squared** | **Predicted**  **R-Squared** | **PRESS** |  |
| Model summary statistics source | | | | | | |
| Linear | 0.31 | 0.2795 | 0.1132 | -0.3716 | 2.33 |  |
| 2FI | 0.22 | 0.7146 | 0.5434 | 0.1019 | 1.52 | Suggested |
| Quadratic | 0.24 | 0.7697 | 0.4736 | -0.6422 | 2.79 |  |
| Cubic | 0.25 | 0.8585 | 0.4342 |  | + | Aliased |
